# 5-Fluorouracil affects assembly of stress granules based on RNA incorporation

**DOI:** 10.1093/nar/gku264

**Published:** 2014-04-09

**Authors:** Christian Kaehler, Jörg Isensee, Tim Hucho, Hans Lehrach, Sylvia Krobitsch

**Affiliations:** 1 Otto Warburg Laboratory, Max Planck Institute for Molecular Genetics, 14195 Berlin, Germany; 2 Department of Biology, Chemistry and Pharmacy, Free University Berlin, 14195 Berlin, Germany; 3 Department of Human Molecular Genetics, Max Planck Institute for Molecular Genetics, 14195 Berlin, Germany; 4 University Hospital Cologne, Department of Anesthesiology and Intensive Care Medicine, Experimental Anesthesiology and Pain Research, 50931 Cologne, Germany; 5 Department of Vertebrate Genomics, Max Planck Institute for Molecular Genetics, 14195 Berlin, Germany; 6 Dahlem Centre for Genome Research and Medical Systems Biology, 14195 Berlin, Germany

## Abstract

The antimetabolite 5-fluorouracil is a widely used chemotherapeutic for the treatment of several solid cancers. However, resistance to 5-fluorouracil remains a major drawback in its clinical use. In this study we report that treatment of HeLa cells with 5-fluorouracil resulted in *de novo* assembly of stress granules. Moreover, we revealed that stress granule assembly under stress conditions as well as disassembly is altered in cells treated with 5-fluorouracil. Notably, we discovered that RACK1, a protein mediating cell survival and apoptosis, is a component of 5-fluorouracil-induced stress granules. To explore the mode of action of 5-fluorouracil accountable for *de novo* stress granule assembly, we analyzed 5-fluorouracil metabolites and noticed that stress granule assembly is caused by RNA, not DNA incorporating 5-fluorouracil metabolites. Interestingly, we observed that other RNA incorporating drugs also cause assembly of stress granules. Thus, our results suggest that incorporation of chemotherapeutics into RNA may result in stress granule assembly with potential significance in chemoresistance.

## INTRODUCTION

Intrinsic or acquired resistance to chemotherapeutics is the major cause for treatment failure and poor prognosis in many cancers. The fluoropyrimidine 5-fluorouracil (5-FU) is widely used in the treatment of a variety of solid cancers, such as head, neck, breast, and colorectal cancer (1). Even so, one major drawback in the clinical application is resistance and non-responding to 5-FU. Additionally, regardless of the broad range of its use over the last five decades, the mode of action is still not completely understood. 5-FU was primarily developed to interfere with the activity of the enzyme thymidylate synthase (2), but incorporation of 5-FU metabolites into DNA and RNA significantly contributes to cytotoxicity (1,3,4). Incorporation of the 5-FU metabolite fluorodeoxyuridine triphosphate (FdUTP) into DNA leads to DNA strand breaks and apoptosis, whereas incorporation of the metabolite fluorouridine triphosphate (FUTP) into RNA results in several defects of the cellular RNA metabolism. Notably, incorporation of FUTP into RNA was discovered as key mechanism conferring cytotoxic effects of 5-FU (5,6). It was shown that 5-FU incorporation into RNA was tremendously higher than into DNA, substantiating that the cytotoxic effects of 5-FU could be probably predominantly RNA-mediated (3). Along this line, inhibition of ribosome biogenesis was linked to 5-FU cytotoxicity (7). Other studies demonstrated that rRNA maturation is an important target for 5-FU (8,9). Interestingly, a yeast screen revealed that yeast strains deficient for genes involved in rRNA and tRNA maturation/modification were particularly 5-FU sensitive, suggesting that tRNA destabilization and defects in rRNA maturation contribute to 5-FU cytotoxicity (8–10). This was further supported by a study using *Schizosaccharomyces pombe* (11). In this light, the ds-RNA-dependent kinase, PKR (protein kinase RNA-activated), has been identified as key target of 5-FU, inducing phosphorylation of the eukaryotic translation initiation factor eIF2α and promoting apoptosis (12). Therefore, it is plausible that resistance to 5-FU might be based on several pathways implicated in the cellular DNA and RNA metabolism.

Over the last years, significant links between stress granule (SG) assembly and resistance to chemotherapeutic-mediated apoptosis were discovered (13–18). SGs are cytoplasmic ribonucleoprotein complexes that store and regulate untranslated mRNA molecules (19). They are induced under various conditions of stress, which lead to the activation of serine/threonine kinases such as PKR, HRI (heme-regulated inhibitor), PERK (PKR-like endoplasmatic reticulum kinase) and GCN2 (general control non-derepressible 2) resulting in phosphorylation of eIF2α. The presence of phosphorylated eIF2α limits the assembly of the ternary eIF2-GTP-tRNA^Met^ complex essential for translation initiation, thereby causing accumulation of a stalled 48S preinitiation complex followed by polysome disassembly. SGs consist of eukaryotic initiation factors, small ribosomal subunits and a number of RNA-binding proteins, of which some like TIA-1 (T-cell-restricted intracellular antigen), TIAR (TIA-1-related) and G3BP (Ras GTPase-activating protein-binding protein 1), based on their aggregation-prone properties, are involved in the primary nucleation step of SG assembly (19–21). Moreover, SGs are found in a functional interplay with processing bodies (P-bodies), cellular sites at which mRNA degradation takes place (22). In addition to the role of SGs in the cellular RNA metabolism, evidence has been provided that SGs also represent central hubs for cell signaling, since numerous cell-signaling proteins are sequestered to SGs (23). In regard to SG assembly and resistance to chemotherapeutic-mediated apoptosis, it is quite interesting that sequestration of the cell-signaling protein RACK1 (receptor for activated C kinase 1) to SGs causes suppression of the stress-responsive MAPK (mitogen-activated protein kinase) pathways and thereby inhibition of apoptotic cascades (14,24). In this light, treatment of cells with the chemotherapeutic bortezomib induces phosphorylation of eIF2α through activation of HRI resulting in SG assembly and resistance to apoptosis as well (18).

Due to the finding that 5-FU activates the protein kinase PKR leading to phosphorylation of eIF2α (12), which is a criterion for SG assembly, we hypothesized that 5-FU could be linked to SG assembly. Here we describe that 5-FU treatment of HeLa cells resulted in *de novo* assembly of SGs. Moreover, we show that 5-FU affects SG assembly under stress conditions as well as disassembly. Remarkably, RACK1, a protein mediating cell survival and apoptosis (14), is sequestered into 5-FU-induced SGs. We further discovered that the induction of SGs by 5-FU correlates with the presence of phosphorylated eIF2α. To understand the mode of action of 5-FU underlying this phenomenon, we finally investigated which intracellular 5-FU metabolite is responsible. We observed that the RNA incorporating 5-FU metabolite, not the DNA incorporating 5-FU metabolite, prompts SG assembly.

## MATERIALS AND METHODS

### Cell culture, drug treatment and stress induction

HeLa, A459, DU145, HEK293T, HepG2, RWPE1 cells or WI38 fibroblasts were cultivated at 37°C and 5% CO_2_ in Dulbecco's modified Eagle's medium (Gibco) supplemented with 10% fetal bovine serum (Biochrom) and 1% penicillin/streptomycin (Biochrom). For drug experiments, cells were treated with different concentrations of 5-FU (Sigma), 5-fluorouridine (FUrd, Sigma) or 5-fluorodeoxyuridine (FdUrd, Sigma) for 72 h or as indicated otherwise. Moreover, combinatorial treatment of 5-FU or FUrd with uridine (Sigma), or sole treatment with 6-thioguanine (Sigma), 5-azacytidine (Sigma), gemcitabine (Selleck) or trifluorothymidine (TFT, Tokyo Chem. Ind.) was performed for 72 h at the indicated concentrations. For stress induction, 5-FU-treated cells were additionally treated with 0.5 mM sodium arsenite (Merck) for 1 h, while control cells were left untreated. For recovery experiments, medium was renewed after drug treatment and cells were incubated for additional 72 h.

### Immunofluorescence and *in situ* hybridization

For immunofluorescence experiments cells were plated at a density of 8000 cells/well in a 24-well plate and treated with the different drugs as indicated. Cells were then washed with phosphate buffered saline (PBS), fixed with 2% formaldehyde for 10 min and ice-cold methanol for 30 min, and incubated with 3% bovine serum albumin (BSA)/PBS for 30 min. Subsequently, cells were incubated with the respective primary antibodies in 3% BSA/PBS for 1 h at room temperature [rabbit anti-ATXN2L (Bethyl A301–370A, 1:300), mouse anti-ATXN2 (BD Biosciences 611378, 1:200), rabbit anti-ATXN2 (Bethyl A301–118A, 1:200), mouse anti-DCP1A (Abnova M06, 1:400), rabbit anti-DCP1A (C-terminus, Sigma D5444, 1:200), rabbit anti-DDX6 (Novus 200–192, 1:300), rabbit anti-P-eIF2α Ser51 (Cell Signaling 3597, 1:100), rabbit anti-eIF4G1 (Abcam 47649, 1:300), mouse anti-G3BP1 (Abnova M01, 1:200), mouse anti-RACK1 (BD Biosciences 610177, 1:200), and mouse anti-TIAR (BD Biosciences 610352, 1:200)]. Afterward, cells were incubated with secondary antibodies for 1 h [goat anti-mouse Alexa Fluor 488 and anti-rabbit Alexa Fluor 594 (Invitrogen, 1:500)]. After washing the cells with PBS, nuclei were stained with Hoechst 33258 (bisbenzimide, Sigma), and samples were mounted with Fluoromount-G (Southern Biotech).

For *in situ* hybridization, cells were fixed with 2% formaldehyde for 10 min and permeabilized with 0.5% Triton X-100/PBS for 10 min. Cells were then incubated with prehybridization buffer (2x saline-sodium citrate (SSC), 20% formamide, 0.2% BSA) for 30 min at 37°C and with prehybridization buffer containing 0.5 nM Cy3-coupled-oligo-dT_30_ (Gentaur 26–4330–02) for 3 h at 37°C. After washing steps with 2x SSC/20% formamide, 2x SSC and 1x SSC for 5 min at room temperature, cells were washed with PBS and further processed for immunofluorescence as described above.

Cells were analyzed using a confocal microscope (LSM 700, Zeiss) on an inverted stand (Axiovert 200 M, Zeiss) using objective Plan-NEOFLUAR 40x 1.3 oil DIC. Images were acquired using Zeiss software ZEN version 5.5.

### Immunoblotting

For investigating the effect of 5-FU, FUrd or FdUrd on eIF2α phosphorylation, cells were treated with different concentrations, washed with PBS and lysed with lysis buffer [20 mM Tris-HCl, 150 mM NaCl, 1 mM EDTA, 1% Triton X-100, 2.5% Protease-inhibitor (‘complete’ tablets, Roche), and 25 U/ml benzonase (Merck)] for 30 min at 4°C. Laemmli's sample buffer containing 0.1 M dithiothreitol (DTT) was added to 50 μg of each lysate. Samples were then incubated at 95°C for 5 min and loaded onto a 10% sodium dodecyl sulphate (SDS) gel. After separation, proteins were transferred to a polyvinylidene fluoride (PVDF) membrane (Millipore) using a semi-dry blotting system (PeqLab). Membranes were then incubated in 2% milk powder and the respective primary antibodies were added [rabbit anti P-eIF2α Ser51 (Cell Signaling 3597, 1:500) and rabbit anti eIF2α (Cell Signaling 9722, 1:500)]. After incubation overnight, membranes were washed and incubated with secondary antibodies in 2% milk powder for 1 h [POD-conjugated goat anti-mouse or anti-rabbit (1:10000, Sigma)]. Finally, proteins were visualized using Western Lightning enhanced chemoluminescence (ECL) solutions (Perkin Elmer). Equal protein loading was confirmed by Coomassie Brilliant Blue (Sigma) staining of a separate SDS gel.

### Quantitative high-content screening microscopy

For quantitative high-content screening (HCS) microscopy analyses cells were plated at a density of 2000 cells/well in a 96-well imaging plate (Greiner μClear) and treated with a concentration gradient of 5-FU as indicated. Cells were immunolabeled as described above and nuclei stained with DAPI (diamidinophenylindole, Sigma). Plates were scanned using a Thermo Fisher Cellomics ArrayScan VTI and images of 512 × 512 pixels were acquired with a 20x objective and analyzed using the Cellomics software package (Colocalization V.4 Bioapplication). Cell nuclei were identified by DAPI staining and according to the object identification parameters size: 100–1200 μm^2^, ratio of perimeter squared to 4π area: 1–2, length-to-width ratio: 1–2, average intensity: 50–1000, total intensity: 3×10^4^–2×10^7^. SGs and P-bodies were identified within a circular region extending the nucleus by maximally 20 μm. The object identification parameters for SGs and P-bodies were 1.5–20 μm^2^, ratio of perimeter squared to 4π area: 1–1.8, length-to-width ratio: 1–1.8, average intensity: 100–1500 and total intensity: 5×10^3^–5×10^4^.

## RESULTS

### SG assembly is induced by 5-FU and altered under stress conditions

**Figure 1.**
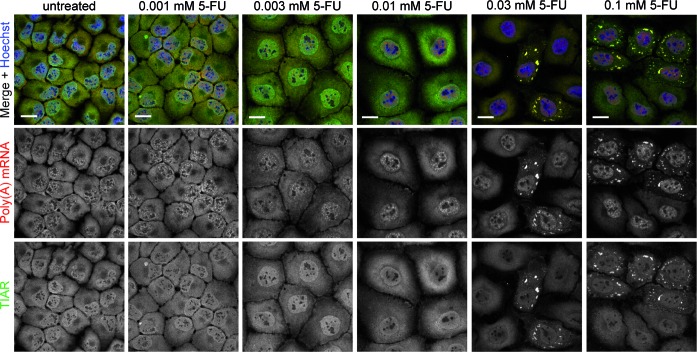
5-FU treatment induces *de novo* assembly of SGs. HeLa cells were treated with increasing concentrations of 5-FU for 72 h as indicated and processed for confocal microscopy to analyze the localization of poly(A) mRNA (red) and the SG marker protein TIAR (green). Nuclei were stained with Hoechst. Scale bars represent 20 μm.

**Figure 2.**
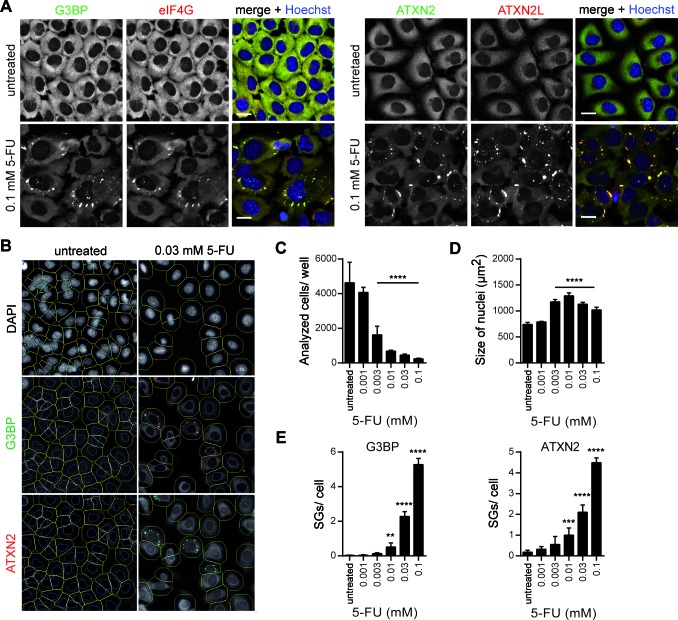
5-FU-induced SGs contain *bona fide* SG marker proteins. **(A)** HeLa cells were treated with 0.1 mM 5-FU for 72 h and processed for immunostaining of the SG marker proteins G3BP (green) and eIF4G (red) (left panel), or ATXN2 (green) and ATXN2L (red) (right panel). Nuclei were stained with Hoechst. Scale bars represent 20 μm. **(B)** Representative view fields of the quantitative HCS microscopy are shown. Outer cell borders (green lines) were calculated by extending the nuclear region (blue lines). G3BP- and ATXN2-positive SGs were quantified. **(C–E)** Cell number **(C)**, size of nuclei **(D)** and number of G3BP- or ATXN2-positive SGs **(E)** were analyzed by HCS and quantified. Results are expressed as mean ± SD from one representative experiment; *n* = 5 replicate wells, *P < 0.05; **P < 0.01; ***P < 0.001, one-way ANOVA with Tukey's Multiple Comparison post test.

5-FU treatment activates PKR in a number of mouse and human cell lines inducing phosphorylation of eIF2α and finally apoptosis (12). Phosphorylation of eIF2α, however, also results in cytoprotection as cell survival pathways are induced, e.g. the formation of SGs (25,26). Therefore, we wanted to directly address the question whether treatment of 5-FU may possibly be related to SG assembly and if, to further explore the underlying mechanism. Since Garcia and colleagues (12) demonstrated that 5-FU treatment resulted in detectable levels of phosphorylated eIF2α at earliest 16 or 24 h post treatment, we reasoned to incubate HeLa cells with different concentrations of 5-FU for up to 24 h and to visualize TIAR for detection of SG assembly. Our microscopic analysis revealed that TIAR-positive foci, however, were absent in cells treated with the lower 5-FU concentrations selected and that only a small number of cells treated with 1 mM 5-FU, the highest concentration used, exhibited TIAR-positive foci after a 16 or 24 h 5-FU treatment (Supplementary Figure S1). For that reason we decided to perform further experiments with different 5-FU concentrations for a prolonged time period of 72 h. Subsequently, cells were prepared for confocal microscopy and SG assembly was visualized by TIAR and poly(A) mRNA staining. As illustrated in Figure 1, we observed that cells treated with 0.03 or 0.1 mM 5-FU exhibited distinct poly(A) mRNA- and TIAR-positive foci, whereas such foci were absent in cells treated with lower 5-FU concentrations or left untreated as control. Please note that the treatment of cells with 1 mM 5-FU used (Supplementary Figure S1) has a considerable effect on cell viability over a time period of 72 h and is therefore inapplicable for our cellular phenotypic analyses (data not shown). To further investigate whether this observation is not only restricted to HeLa cells, we selected a panel of different cell lines for further analyses and observed TIAR-positive foci in all 5-FU-treated cell lines (Supplementary Figure S2). Next, we included other SG marker proteins in our analysis. For this, we decided to treat HeLa cells with 0.1 mM 5-FU for 72 h, a condition, at which the percentage of HeLa cells exhibiting poly(A) mRNA- and TIAR-positive foci was most significant compared to the other 5-FU concentration used (Figure 1), and visualized the SG marker proteins G3BP, eIF4G, ATXN2 (Ataxin-2), and ATXN2L (Ataxin-2-like). Indeed, 5-FU-treated cells displayed distinct foci that were positive for all analyzed SG marker proteins (Figure 2A). Moreover, we quantified these observations using a HCS microscopy approach recently established by us (27). The system automatically acquires images of stained cells in multi-well plates. Cells are identified by image analysis according to their nuclear DAPI staining and fixed object selection parameters (see Materials and Methods section for details). SGs are then quantified within a circular area extending the nuclear region (Figure 2B). As expected for a cytostatic compound, 5-FU dose-dependently inhibited cell proliferation (Figure 2C). In addition, nuclei were significantly enlarged upon 5-FU treatment peaking at 0.01 mM (Figure 2D), a concentration reducing cell number to ∼20%. Of note, enlargement of nuclei was reduced at higher 5-FU doses, representing concentrations that induce SG assembly. In line with our confocal microscopy studies, we observed a dose-dependent increase of SGs per cell using G3BP and ATXN2 as marker proteins (Figure 2E), demonstrating that 5-FU induces *de novo* SG assembly under the chosen conditions.

**Figure 3.**
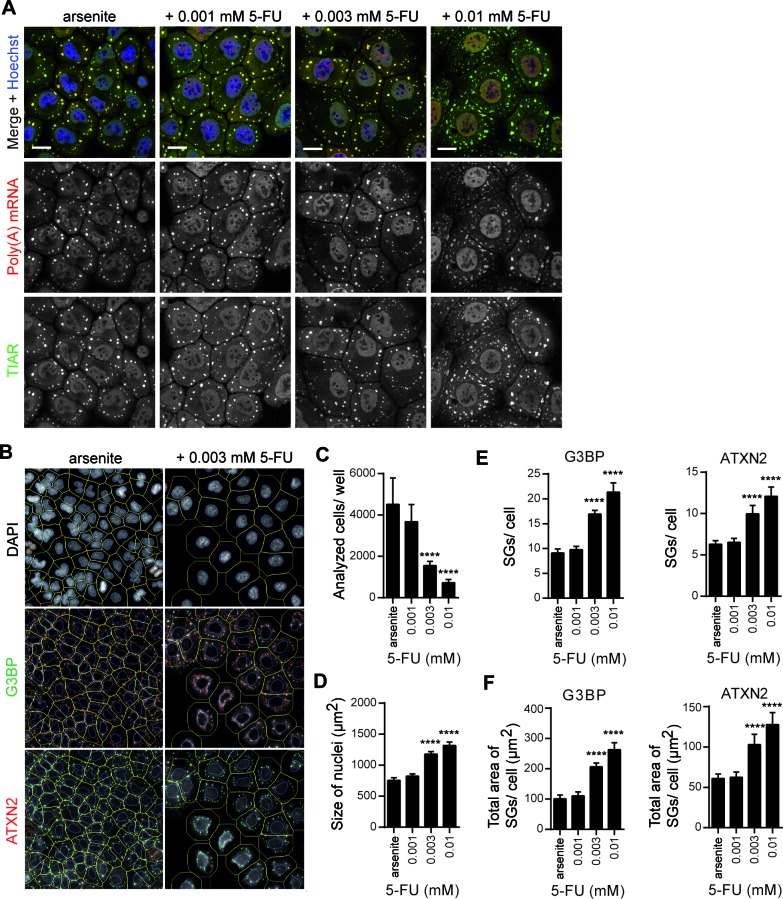
5-FU treatment enhances SG assembly under oxidative stress conditions. **(A)** HeLa cells were treated with increasing concentrations of 5-FU for 72 h, exposed to 0.5 mM arsenite for 1 h and processed to detect poly(A) mRNA (red), and the SG marker protein TIAR (green). Nuclei were stained with Hoechst. Scale bars represent 20 μm. **(B)** Representative view fields of the quantitative HCS microscopy analysis are shown. Outer cell borders (green lines) were calculated by extending the nuclear region (blue lines). G3BP- and ATXN2-positive SGs were quantified. **(C–F)** Cell number **(C)**, size of nuclei **(D)** and number and area of G3BP- or ATXN2-positive SGs **(E, F)** were analyzed and quantified by HCS microscopy. Results are expressed as mean ± SD from one representative experiment; *n* = 5 replicate wells, *P < 0.05; **P < 0.01; ***P < 0.001, one-way ANOVA with Tukey's Multiple Comparison post test.

Since cells encounter cellular stress in a number of human pathologies, we finally set out to investigate whether 5-FU treatment has an effect on SG assembly under stress conditions. We therefore treated HeLa cells with different concentrations of 5-FU, induced cellular stress with arsenite, and then analyzed SG assembly by visualizing poly(A) mRNA and the SG marker protein TIAR. As illustrated in Figure 3A, HeLa cells treated with 0.003 mM or 0.01 mM 5-FU, and arsenite, respectively, exhibited higher numbers of SGs compared to control cells and cells treated with 0.001 mM 5-FU and arsenite. Moreover, the morphology of SGs in 5-FU/arsenite-treated cells was altered, since these appeared to be enlarged in size. These qualitative observations were confirmed by quantitative HCS microscopy (Figure 3B–F). Again, we observed that cell proliferation of 5-FU/arsenite-treated cells was dose-dependently inhibited (Figure 3C), and that nuclei were significantly enlarged (Figure 3D). Cells treated with 0.01 mM 5-FU and arsenite showed almost 2-fold higher numbers of G3BP- and ATXN2-positive SGs compared to cells treated with 0.001 mM 5-FU and arsenite or control cells treated only with arsenite (Figure 3E). Moreover, the total area of SGs measured was increased indicating that the size of SGs was enlarged (Figure 3F). Thus, 5-FU induces *de novo* assembly of SGs and affects SG assembly under stress conditions as well.

### 5-FU-induced SGs sequester RACK1 and display altered disassembly properties

**Figure 4.**
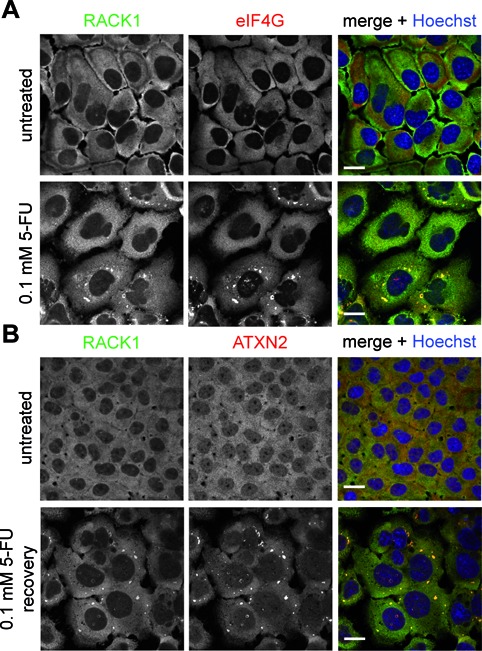
5-FU-induced SGs sequester RACK1 and exhibit altered disassembly properties. **(A)** HeLa cells were treated with 0.1 mM 5-FU for 72 h and **(B)** subsequently recovered from 5-FU treatment for additional 72 h. Localization of RACK1 (green) and eIF4G (red) in **(A)** or RACK1 (green) and ATXN2 (red) in **(B)** was studied. Nuclei were stained with Hoechst. Scale bars represent 20 μm.

A link between SG assembly and resistance to chemotherapeutic-mediated apoptosis was discovered, which is based on the sequestration of the mediator protein RACK1 into SGs. This has a negative impact on the stress-activated p38 and JNK (c-Jun N-terminal kinase)/ MAPK pathways suppressing apoptosis and conferring cytoprotection (14). Accordingly, we investigated whether 5-FU-induced SGs contain RACK1. For this purpose, HeLa cells were treated with 0.1 mM 5-FU, prepared for immunostaining and analyzed by confocal microscopy. Distinct RACK1-positive foci were detected in 5-FU-treated cells that were also labeled for eIF4G (Figure 4A), demonstrating that RACK1 is a component of 5-FU-induced SGs under the chosen experimental conditions.

Since disassembly of SGs and therefore release of RACK1 or other cell-signaling proteins take part in cell regulation as well (14,23,28,29), we lastly decided to include this aspect in our analyses. HeLa cells were therefore treated with 0.1 mM 5-FU for 72 h. Subsequently, the cells were washed and incubated in fresh cell culture medium. Interestingly, we observed that RACK1-positive SGs were quite stable and still detectable in cells after 72 h of recovery (Figure 4B), whereas SG disassembly normally occurs 90–120 min after stress release (30). Thus, 5-FU-induced SGs contain the mediator protein RACK1 and disassembly properties of 5-FU-induced SGs are altered.

### Induction of SGs by 5-FU correlates with phosphorylation of eIF2α

**Figure 5.**
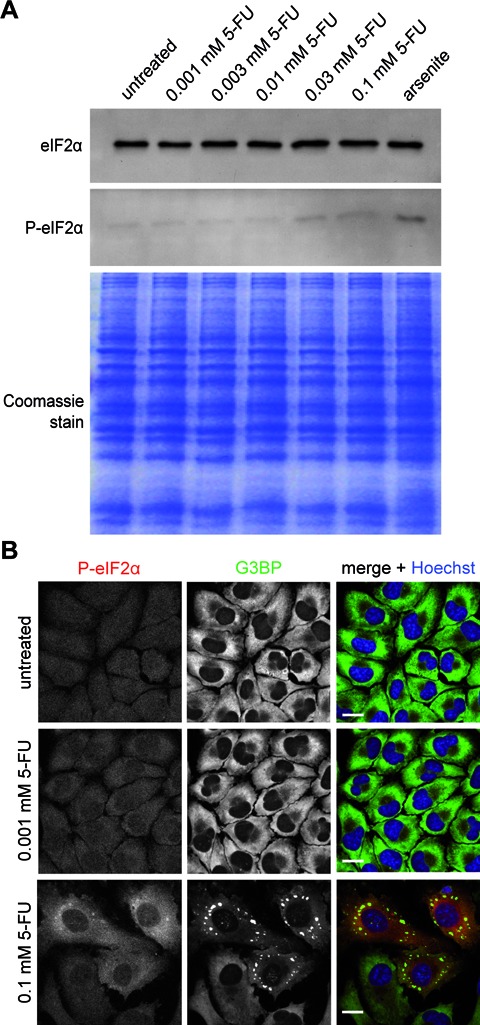
SG assembly induced by 5-FU correlates with phosphorylation of eIF2α. **(A)** HeLa cells were treated with increasing concentrations of 5-FU for 72 h. As a control for eIF2α phosphorylation cells were treated with 0.5 mM arsenite for 1 h. Cell lysates were prepared and intracellular levels of eIF2α and P-eIF2α were analyzed. Equal loading was verified by Coomassie gel staining. **(B)** HeLa cells were treated with increasing concentrations of 5-FU for 72 h and processed for immunostaining of phosphorylated eIF2α (red) and the SG marker protein G3BP (green). Nuclei were stained with Hoechst. Scale bars represent 20 μm.

SG assembly can occur in a eIF2α phosphorylation-dependent and eIF2α phosphorylation-independent manner (31,32). As aforementioned, Garcia and colleagues (12) demonstrated that PKR is activated by 5-FU facilitating phosphorylation of eIF2α. Accordingly, we next set out to investigate whether phosphorylation of eIF2α might occur under our chosen experimental conditions as well. For this, HeLa cells were treated with different concentrations of 5-FU and protein lysates were prepared for western blot analysis. A positive signal for phosphorylated eIF2α was detected if a concentration of 0.03 or 0.1 mM 5-FU was applied to cells (Figure 5A), which is in correlation with SG appearance observed earlier (Figure 1); only a faint signal was observed for lower 5-FU concentrations that was equivalent to the signal detected for untreated HeLa cells. To end with, we performed microscopy to examine whether SG assembly correlates with eIF2α phosphorylation at single cell level using 0.1 mM 5-FU, the concentration that gave rise to a robust SG induction (Figure 1). As controls, we used cells treated with 0.001 mM 5-FU or left cells untreated. As shown in Figure 5B, cells treated with 0.1 mM 5-FU exhibited G3BP-positive foci indicative of SG assembly and were positive for phosphorylated eIF2α compared to 0.001 mM treated 5-FU cells, in which SGs were not induced, or untreated control cells. Thus, SG assembly induced by 5-FU corresponds to the presence of phosphorylated eIF2α.

### The RNA, not DNA incorporating 5-FU metabolite, triggers SG assembly

**Figure 6.**
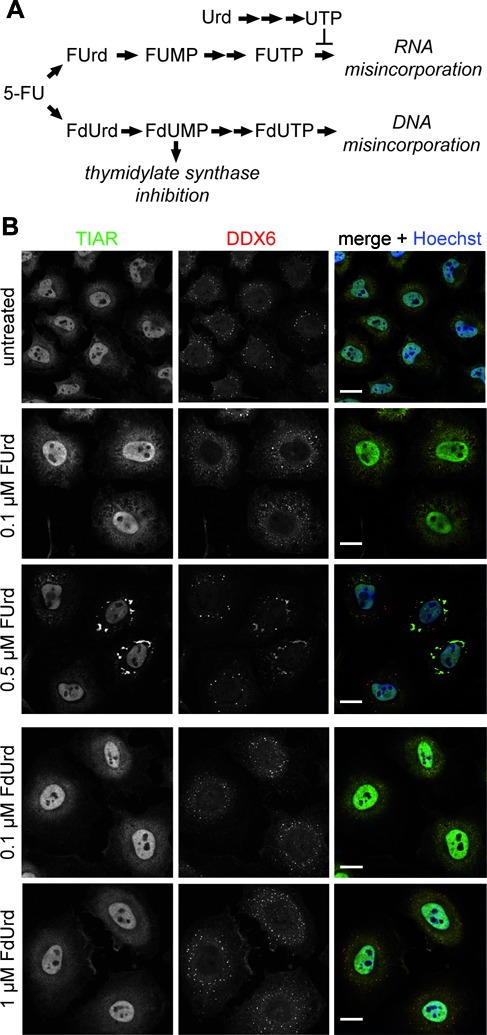
5-FU-induced SG assembly depends on RNA incorporation. **(A)** Schematic representation of the cellular 5-FU metabolism leading to incorporation of the different metabolites into RNA or DNA. **(B)** HeLa cells were treated with two concentrations of the 5-FU metabolites FUrd or FdUrd for 72 h. SG marker protein TIAR (green) and SG/P-body marker protein DDX6 (red) were visualized. Nuclei were stained with Hoechst. Scale bars represent 20 μm.

As illustrated in Figure 6A, after cellular uptake 5-FU is converted into the metabolites FUrd and FdUrd that are further metabolized to FUTP and FdUTP, respectively (1). In addition to the inhibition of thymidylate synthase by FdUMP, the metabolite FdUTP incorporates into DNA causing strand breaks and apoptosis (33). In contrast, FUTP incorporates into RNA representing a key mechanism conferring cytotoxic effects of 5-FU (1). To further dissect which 5-FU metabolite is responsible for the induction of *de novo* SG assembly, HeLa cells were treated with different concentrations of each metabolite that do not affect cell viability (data not shown). Subsequently, cells were prepared for confocal microscopy, imaging the SG marker protein TIAR. Due to the close interplay between SGs and P-bodies (22), we decided at this point to include a marker protein for both SGs and P-bodies, such as DDX6 (DEAD-box protein 6), to detect potential effects on these cellular structures as well. As shown in Figure 6B, HeLa cells treated with 0.5 μM FUrd exhibited TIAR-positive foci that primarily co-localized with DDX6-positive foci, whereas no cytoplasmic TIAR-positive foci were detectable in 0.1 μM FUrd-treated or untreated cells. We also treated cells with two different concentrations of the 5-FU metabolite FdUrd, known to incorporate into DNA. As shown in Figure 6B, no cytoplasmic TIAR-positive foci were detectable indicating that incorporation into RNA rather than DNA is causative for SG assembly.

**Figure 7.**
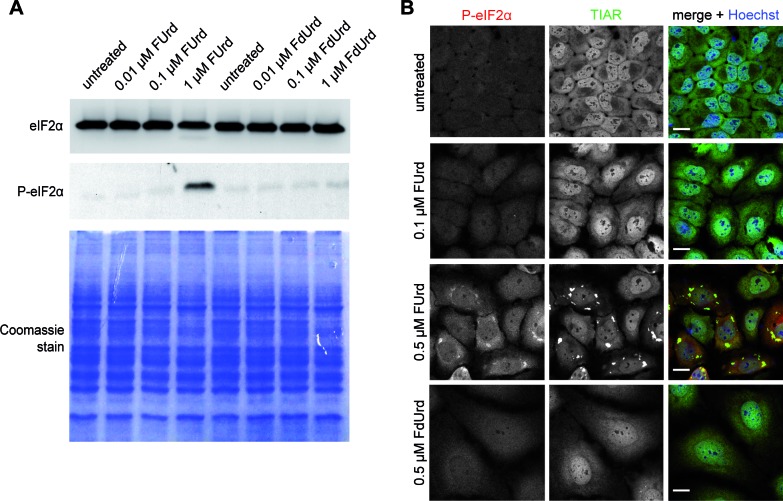
The RNA incorporating 5-FU metabolite induces phosphorylation of eIF2α. **(A)** HeLa cells were treated with increasing concentrations of the 5-FU metabolites FUrd or FdUrd for 72 h. Cell lysates were prepared and intracellular levels of eIF2α and P-eIF2α were analyzed. Equal loading was verified by Coomassie gel staining. **(B)** HeLa cells were treated with the indicated concentrations of FUrd or FdUrd for 72 h and processed for immunostaining of phosphorylated eIF2α (red) and the SG marker protein TIAR (green). Nuclei were stained with Hoechst. Scale bars represent 20 μm.

**Figure 8.**
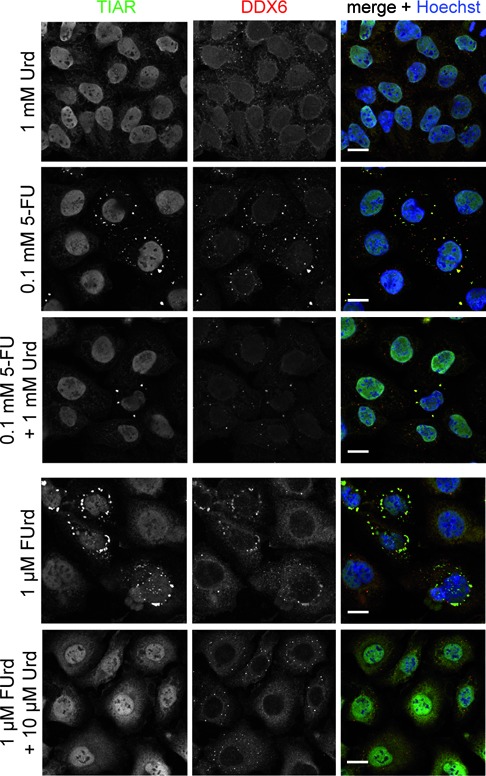
5-FU-induced SG assembly is rescued by interfering with RNA incorporation. HeLa cells were treated with 1 mM Urd, 0.1 mM 5-FU or both, as well as with 1 μM FUrd or a combination of 1 μM FUrd and 10 μM Urd. Localization of SG marker protein TIAR (green) and SG/P-body marker protein DDX6 (red) was investigated. Nuclei were stained with Hoechst. Scale bars represent 20 μm.

In the next step, we investigated whether SG assembly induced by FUrd corresponds to the presence of phosphorylated eIF2α. Again, HeLa cells were treated with different concentrations of FUrd. As controls, cells were treated with the 5-FU metabolite FdUrd, not inducing SG assembly, or cells were left untreated. Subsequently, protein lysates were prepared for western blot analysis. As shown in Figure 7A, a strong signal for phosphorylated eIF2α was observed for protein lysates prepared from FUrd-treated cells, whereas a weak signal was observed if cells were treated with FdUrd, indicating that phosphorylation of eIF2α is triggered by the RNA incorporating 5-FU metabolite, which is in accordance with the presence of SGs (Figure 6B). This observation was also corroborated by our microscopic analysis demonstrating that cells exhibiting FUrd-induced SGs are also positive for phosphorylated eIF2α (Figure 7B). To finally determine whether the observed altered disassembly properties of 5-FU-induced SGs are caused by the RNA incorporating pathway, we additionally carried out recovery experiments, and observed that the disassembly of FUrd-induced SGs is affected (Supplementary Figure S3). To end with, we performed competition experiments using uridine (Urd), which leads to incorporation of uridine triphosphate instead of FUTP into RNA. First we identified a uridine concentration that did not result in induction of SGs (data not shown). Interestingly, we observed that co-treatment of HeLa cells with 0.1 mM 5-FU, the concentration robustly inducing SGs, and 10-fold excess of Urd (1 mM) causes a drop in cells displaying TIAR-positive foci which are additionally less in number compared to cells treated with 5-FU alone (Figure 8). Most importantly, co-treatment of HeLa cells with FUrd and Urd also reduced the number of HeLa cells exhibiting TIAR-positive foci (Figure 8), demonstrating that RNA incorporation is the source inducing SG assembly.

**Figure 9.**
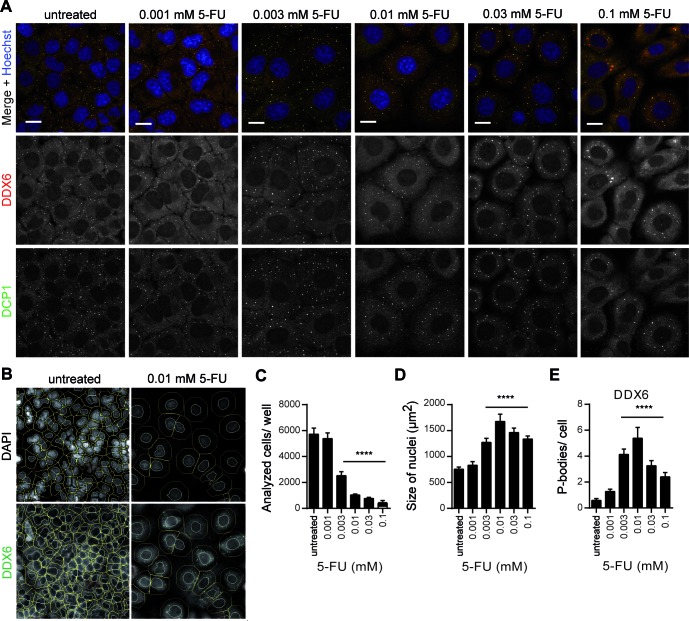
5-FU treatment increases the number of P-bodies. **(A)** HeLa cells were treated with the indicated concentrations of 5-FU for 72 h. The SG/P-body marker protein DDX6 (red) and the P-body marker protein DCP1 (green) were visualized and analyzed by confocal microscopy. Nuclei were stained with Hoechst. Scale bars represent 20 μm. **(B)** Representative view fields of the quantitative HCS microscopy analysis are shown. Outer cell borders (green lines) were calculated by extending the nuclear region (blue lines). DDX6-positive P-bodies were quantified. **(C–E)** Cell number **(C)**, size of nuclei **(D)** and number of DDX6-positive P-bodies **(E)** were analyzed by HCS microscopy. Results are expressed as mean ± SD from one representative experiment; *n* = 5 replicate wells, *P < 0.05; **P < 0.01; ***P < 0.001, one-way ANOVA with Tukey's Multiple Comparison post test.

Looking at concentrations at which 5-FU or the RNA incorporating 5-FU metabolite FUrd did not induce SGs (Figure 6B), we reasoned from the observed DDX6-positive foci that 5-FU and its metabolites seem to affect P-bodies as well. To further substantiate this first impression, we treated cells with 5-FU and stained in addition to DDX6, a marker protein for both SGs and P-bodies, DCP1 (mRNA decapping enzyme 1), a robust P-body marker (34), for our microscopic analysis. As shown in Figure 9, 5-FU-treated cells displayed an increase in DCP1-positive foci that co-localized with DDX6-positive foci primarily if a lower 5-FU concentration of 0.003 mM 5-FU was applied, a concentration that did not significantly induce SG assembly (Figure 2E). Finally we quantified the effect of 5-FU by HCS microscopy as described, and detected a dose-dependent increase in P-body number at this concentration. Further analysis with both 5-FU metabolites suggested that the RNA and the DNA incorporating pathway might affect the cellular P-body number (Supplementary Figure S4). Thus, these findings demonstrate that 5-FU treatment also has an effect on P-bodies.

### Incorporation of chemotherapeutics into RNA is responsible for SG assembly

**Figure 10.**
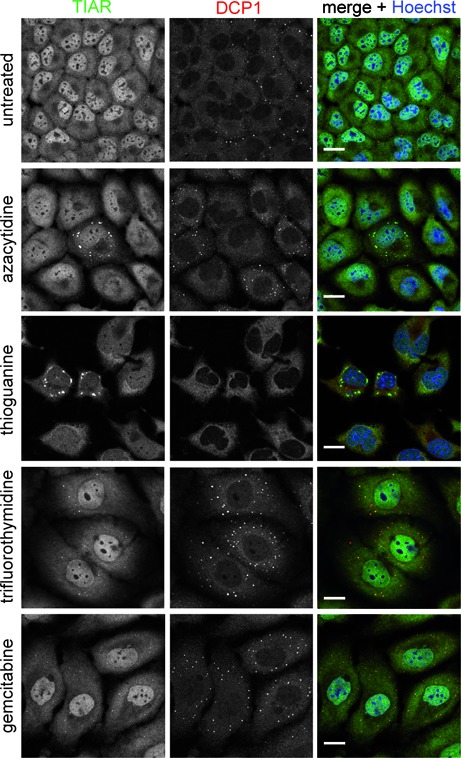
RNA incorporating drugs induce SG assembly. HeLa cells were treated with the RNA incorporating agents 5-azacytidine (50 μM) and 6-thioguanine (10 μM), or the DNA incorporating agents trifluorothymidine (10 μM) and gemcitabine (100 nM) for 72 h. Subsequently, the cellular localization of the SG marker protein TIAR (green) and the P-body marker protein DCP1 (red) was analyzed. Nuclei were stained with Hoechst. Scale bars represent 20 μm.

To finally investigate whether incorporation of drugs into RNA could be a general mechanism inducing SG assembly, we tested other chemotherapeutics known to incorporate into RNA, such as 5-azacytidine and 6-thioguanine (35,36), or as control, chemotherapeutics known to incorporate mainly into DNA, such as trifluorothymidine (TFT) or gemcitabine (37,38). We observed that HeLa cells treated with 5-azacytidine and 6-thioguanine displayed TIAR-positive foci, whereas HeLa cells treated with TFT or gemcitabine did not exhibit such foci even with higher concentrations applied (Figure 10; data not shown). In addition, we included DCP1 in our analysis to further verify the observation made for 5-FU in regard to P-bodies. We observed that cells treated with the DNA incorporating chemotherapeutics displayed a clear increase in the number of DCP1-positive foci in contrast to the RNA incorporating chemotherapeutics. Thus, our results demonstrate that incorporation of chemotherapeutics into RNA and DNA might affect P-bodies, whereas RNA incorporating agents most likely trigger SG assembly.

## DISCUSSION

Over the past years evidence has been provided that induction of SG assembly attributable to chemotherapeutics applied and resistance to apoptotic cascades is interconnected, representing a central task in cancer biology. The major drawback in the clinical use of 5-FU is resistance and non-responding (1). Interestingly, the protein kinase PKR has been identified as key target for 5-FU promoting apoptosis (12). Nevertheless, PKR activity also leads to phosphorylation of eIF2α inducing SG assembly thereby facilitating the cellular potential to counteract 5-FU-induced cytotoxicity. In this perspective, we demonstrated here that 5-FU treatment of HeLa cells resulted in *de novo* assembly of SGs, which contain the core SG proteins TIAR and G3BP as well as poly(A) mRNA. Moreover, induction of SGs by 5-FU correlates with the presence of phosphorylated eIF2α. To understand the mode of action of 5-FU underlying, we investigated which intracellular 5-FU metabolite is responsible for this finding. We discovered that the 5-FU metabolite FUrd that is further converted into FUTP and incorporated into RNA prompts SG assembly. We finally suggest that incorporation of drugs into RNA could be a prevalent underlying mechanism inducing *de novo* SG assembly, since we observed that other drugs known to incorporate into RNA also have the potential of inducing SGs.

Several lines of evidence have been provided demonstrating that induction of SGs by drugs used in chemotherapy is likely based on different cellular pathways. Moreover, it is also quite plausible that mammalian cells are capable of assembling two types of SGs: one being cytoprotective and the other inhibiting cell survival. As described earlier, the chemotherapeutic drug bortezomib, an inhibitor of the 26S proteasome, induces SG assembly, which is dependent upon the activity of HRI and phosphorylation of eIF2α (18). Notably, HRI deficiency inhibits SG assembly enabling bortezomib-induced apoptosis (18). In line with this, mislocalization of p21 mRNA into SGs through its association with the SG component CUGBP1 (CUG triplet repeat RNA-binding protein 1) resulted in resistance to bortezomib-induced apoptosis, whereas p21 or CUGBP1 depletion sensitizes cells to bortezomib-induced apoptosis (39). Interestingly, oxaliplatin, known to induce the DNA damage response, led to PERK activation and eIF2α phosphorylation and SG assembly (13). As outlined before, the sequestration of the cell-signaling protein RACK1 to SGs suppresses the stress-responsive MAPK pathways therefore inhibiting apoptotic cascades (14). Furthermore, the oncogenic mTORC1–eIF4E pathway promotes the assembly of SGs with antiapoptotic properties (40). As further shown, the inactivation of the mTOR (mammalian target of rapamycin) pathway prevents SG assembly blocking the SG-associated antiapoptotic p21 pathways (40). On the other hand, darinaparsin, an organic arsenical, induces formation of SGs through inhibition of microtubule polymerization; however, SG formation is incomplete as smaller and diffuse SGs were observed, a cellular condition that might result in apoptosis (17). Recently, sodium selenite, a selenium compound with chemotherapeutic properties, was shown to induce assembly of SGs as well (15). Notably, phosphorylation of eIF2α is not mandatory for the assembly of selenite-induced SGs but contributes to it. Here, disruption of the eIF4F complex through 4EBP1 by selenite takes part in SG assembly. Notably, selenite-induced SGs did not comprise RACK1 and possess cytotoxic properties, since accumulation of reactive oxygen species (ROS) was observed under selenite treatment. Interestingly, a combinatorial treatment of selenite and arsenite gave rise to reduced SGs compared to selenite and arsenite treatment alone (15). We observed in our study that 5-FU-induced SGs contain RACK1, which mediates between apoptotic and cytoprotective cellular pathways (14). Moreover, we discovered that SG assembly was triggered by 5-FU and arsenite treatment. Further detailed studies are necessary to dissect whether these SGs are indeed cytoprotective and which downstream signaling pathways are further affected. Since we observed that 5-FU has an effect on SG disassembly as well, this will be another interesting perspective to follow up.

Another interesting observation was the fact that the number of P-bodies was affected by 5-FU as well as by both metabolites applied, while only RNA incorporating chemotherapeutics induced SG assembly. Interestingly, selenite treatment might affect the P-body number as well; however, this finding was statistically not significant (15). Recently, P-bodies have been implicated in the cellular response to hypoxic stress. The translation of the hypoxia inducible factor HIF-1α, the master regulator of the cell to hypoxic conditions, is regulated by the P-body component DDX6 (41). Hypoxia is a common cellular condition of solid tumors also inducing SGs (42). Further investigations are required to understand the mechanisms underlying 5-FU induction of P-bodies and its potential significance in cancer biology and chemoresistance.

## SUPPLEMENTARY DATA

Supplementary Data are available at NAR Online.

SUPPLEMENTARY DATA
