# Supplementary material for: 5-Fluorouracil affects assembly of stress granules based on RNA incorporation
Source: Nucleic Acids Res. 2014 Apr 9;42(10):6436–47. doi: 10.1093/nar/gku264 (PMC4041438; doi:10.1093/nar/gku264)
Supplement: SUPPLEMENTARY DATA [file supp_42_10_6436__index.html]

5-Fluorouracil affects assembly of stress granules based on RNA incorporation — 5-Fluorouracil affects assembly of stress granules based on RNA incorporation — SUPPLEMENTARY DATA 

# 5-Fluorouracil affects assembly of stress granules based on RNA incorporation

## SUPPLEMENTARY DATA

**Files in this Data Supplement:**

- Supplementary Data
