# Supplementary material for: 5-Fluorouracil affects assembly of stress granules based on RNA incorporation
Source: Nucleic Acids Res. 2014 Apr 9;42(10):6436–47. doi: 10.1093/nar/gku264 (PMC4041438; doi:10.1093/nar/gku264)
Supplement: SUPPLEMENTARY DATA [file supp_gku264_nar-00119-v-2014-File012.pdf]

5-Fluorouracil affects assembly of stress granules based on RNA incorporation

Christian Kaehler, Jörg Isensee, Tim Hucho, Hans Lehrach and Sylvia Krobitsch

Figure S1. Time-course of 5-FU-induced SG assembly. HeLa cells were treated with increasing concentrations of 5-FU for the indicated time periods and processed for immunostaining of the SG marker protein TIAR. Nuclei were stained with Hoechst. Scale bars represent 20 µm.

Figure S2. 5-FU induction of SGs is a general cellular phenomenon. DU145, RWPE-1, A549, HepG2, HEK293T, or WI-38 cells were treated with the indicated 5-FU concentrations for 72 h. Afterward, the SG marker protein TIAR was visualized and analyzed by microscopy. Nuclei were stained with Hoechst. Scale bars represent 20 µm.

Figure S3. SGs induced by the RNA incorporating 5-FU metabolite sequester RACK1 and display altered disassembly properties. HeLa cells were treated with 0.5 µM FUrd for 72 h or subsequently recovered from FUrd treatment for additional 72 h. As control HeLa cells were treated with 0.5 mM arsenite for 1 h and subsequently recovered for 120 min. Localization of RACK1 (green) and ATXN2 (red) was studied. Nuclei were stained with Hoechst. Scale bars represent 20 µm.

Figure S4. 5-FU metabolites have an effect on P-bodies. HeLa cells were treated with increasing concentrations of the 5-FU metabolites **(A)** FUrd or **(B)** FdUrd for 72 h, DDX6 (red) and DCP1 (green) were visualized and analyzed by confocal microscopy. Nuclei were stained with Hoechst. Scale bars represent 20 µm.

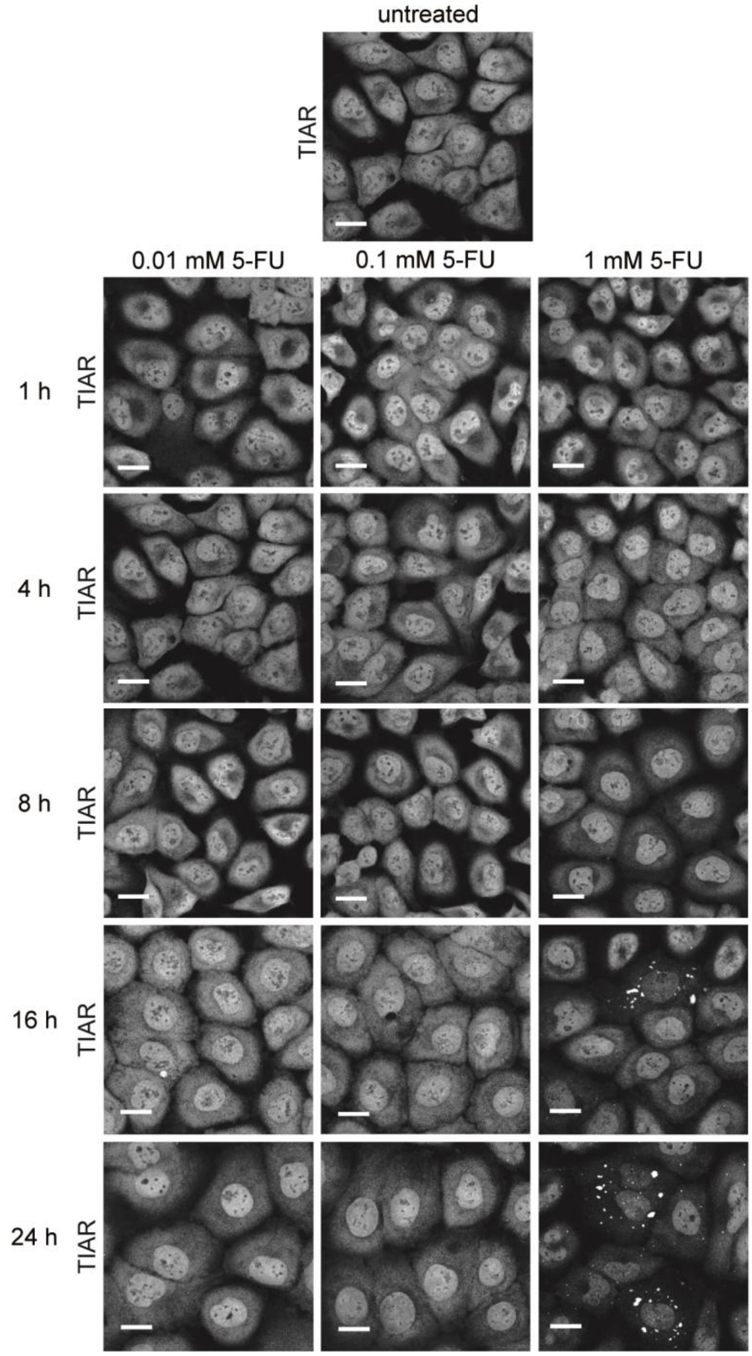
untreated
TIAR
0.01 mM 5-FU
0.1 mM 5-FU
1 mM 5-FU
1 h
4 h
8 h
16 h
24 h

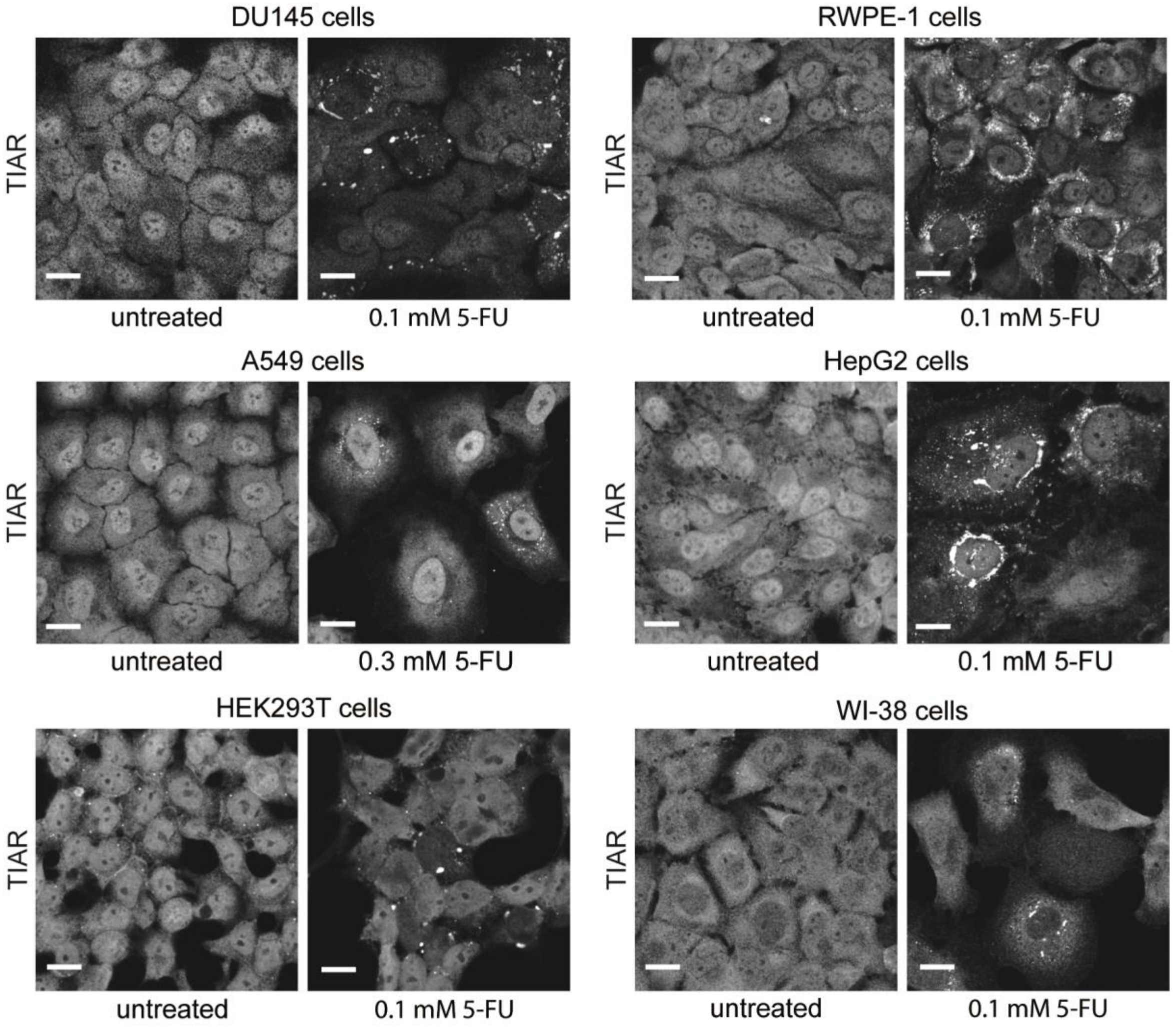
DU145 cells
RWPE-1 cells
TIAR
untreated
0.1 mM 5-FU
A549 cells
HepG2 cells
0.3 mM 5-FU
HEK293T cells
WI-38 cells

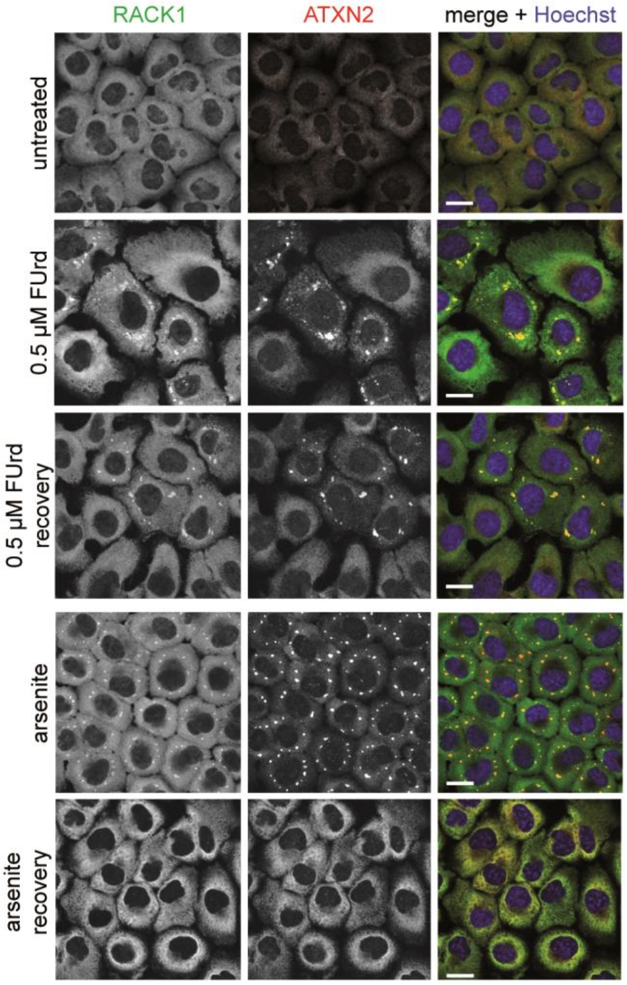
RACK1
ATXN2
merge + Hoechst
untreated
0.5 µM FUrd
0.5 µM FUrd recovery
arsenite
arsenite recovery

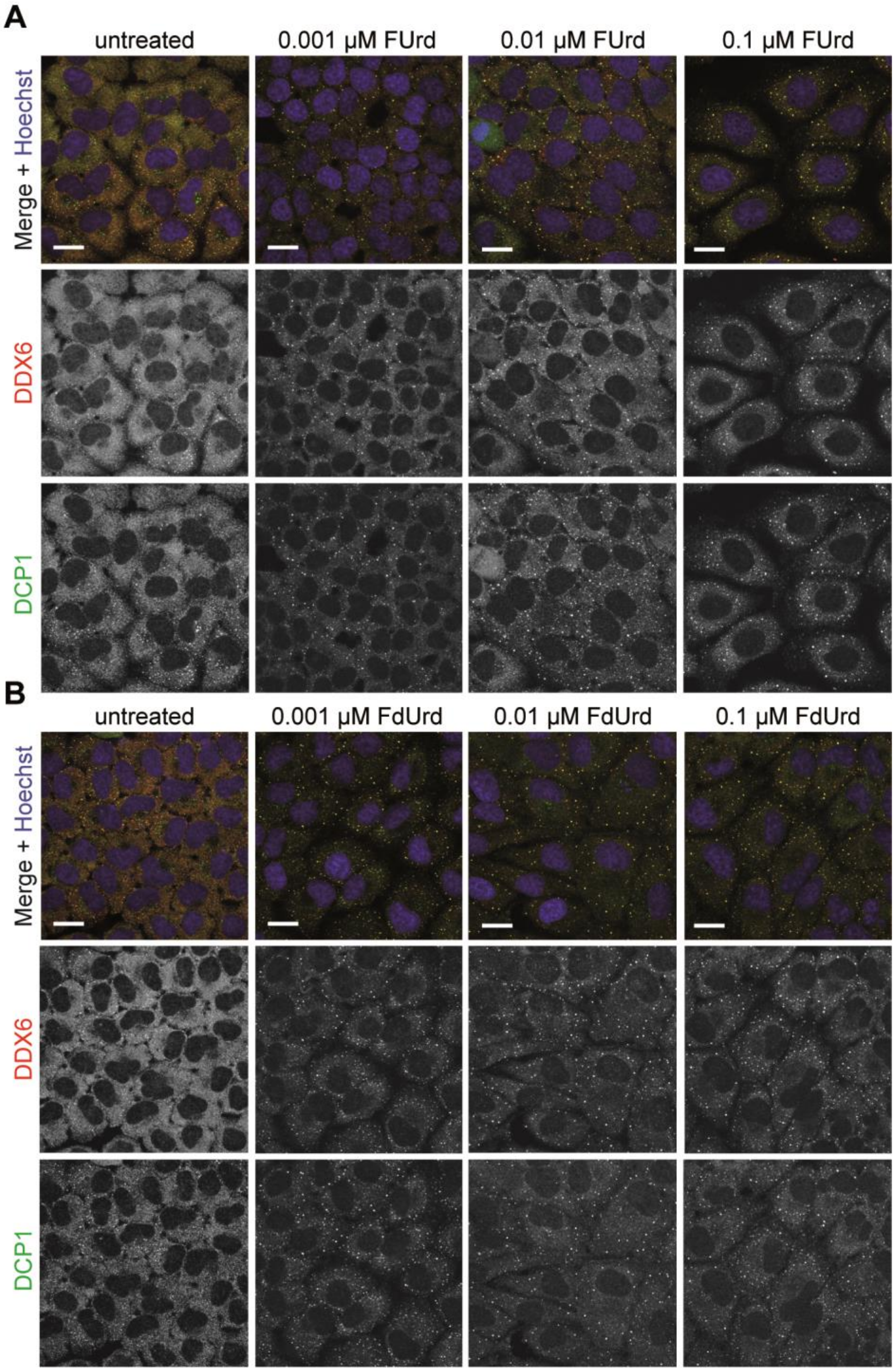

A
untreated
0.001 µM FUrd
0.01 µM FUrd
0.1 µM FUrd
Merge + Hoechst
DDX6
DCP1
B
untreated
0.001 µM FdUrd
0.01 µM FdUrd
0.1 µM FdUrd
Merge + Hoechst
DDX6
DCP1
